# The Association of Human Parvovirus B19 Infection on the Course of Vietnamese Patients with Rheumatoid Arthritis

**DOI:** 10.3390/medicina61091546

**Published:** 2025-08-28

**Authors:** Trieu Van Manh, Mai Ly Thi Nguyen, Ngo Thu Hang, Ngo Truong Giang, Can Van Mao, Luu Thi Binh, Nguy Thi Diep, Bui Tien Sy, Tran Thi Thanh Huyen, Vu Nhi Ha, Le Duy Cuong, Khac Cuong Bui, Hoang Van Tong, Nguyen Linh Toan

**Affiliations:** 1 Department of Pathophysiology, Vietnam Military Medical University, Hanoi 100000, Vietnam; manhytn@gmail.com (T.V.M.); drngohang1986@gmail.com (N.T.H.); canvanmao@vmmu.edu.vn (C.V.M.); buikhaccuong@gmail.com (K.C.B.); hoangvantong@vmmu.edu.vn (H.V.T.); 2 Department of Internal Medicine, Thai Nguyen University of Medicine and Pharmacy, Thai Nguyen 250000, Vietnam; luuthibinh@tump.edu.vn; 3 Department of Biochemistry, Military Hospital 103, Vietnam Military Medical University, Hanoi 100000, Vietnam; dr.nguyenmaily@gmail.com; 4 Department of Biology and Medical Genetics, Vietnam Military Medical University, Hanoi 100000, Vietnam; legiangngo@gmail.com; 5 Hanoi Nephrology Hospital, Hanoi 100000, Vietnam; 6 Vietnamese-German Center for Medical Research (VG-CARE), 108 Military Central Hospital, Hanoi 100000, Vietnam; sybt@benhvien108.vn (B.T.S.); tranthithanhhuyen@gmail.com (T.T.T.H.); 7 Department of Microbiology, Thai Nguyen University of Medicine and Pharmacy, Thai Nguyen 250000, Vietnam; vunhiha.dhytn@gmail.com; 8 Department of Experimental Medicine, 108 Military Central Hospital, Hanoi 100000, Vietnam

**Keywords:** Human parvovirus B19, rheumatoid arthritis, B19V genotype, B19V

## Abstract

*Background and Objectives*: Rheumatoid arthritis (RA) is a systemic autoimmune inflammatory disease, and progressive arthritis is its primary clinical manifestation. The role of human parvovirus B19 (B19V) infection in the progression of RA remains unclear. This study aims to investigate the association between B19V infection and viral genetic distribution in Vietnamese RA patients. *Materials and Methods*: 115 Vietnamese RA patients and 86 healthy controls (HCs) were enrolled in this observational study at the Thai Nguyen National Hospital from January 2019 to December 2021. B19V DNA was examined in serum and synovial fluid samples from RA patients using nested PCR and real-time PCR. B19V antibodies were detected in serum samples using ELISA. *Results*: B19V DNA was detected in the serum of 2 out of 115 (1.74%) RA patients but not in any HCs. Interestingly, B19V DNA was present in 12 out of 68 (17.65%) RA patients with knee effusion in their synovial fluid. Anti-B19V-IgG and anti-B19V-IgM were detected in the serum of 42.61% and 2.61% of RA patients, respectively, and in 24.42% and 12.79% of HCs, respectively. Anti-B19V-IgG levels were significantly higher in the serum of RA patients than in the serum of HCs (*p* = 0.007). However, anti-B19V-IgM was more commonly detected in HC serum than in RA patient serum (*p* = 0.006). Phylogenetic analysis showed that all B19V strains belonged to genotype 1 and subgenotype 1A. *Conclusions*: B19V infection is frequent in RA patients and suggests a contribution of B19V to the progression of RA, particularly in a B19V genotype-1- and subgenotype-1A-dependent manner and emphasises the need for early detection and management of B19V infection in RA patients.

## 1. Introduction

Rheumatoid arthritis (RA), a prevalent systemic autoimmune inflammatory disease, primarily targets the joints, leading to progressive disability, premature death, and high socioeconomic costs [1]. In 2019, the estimated incidence of RA was 18.5 million cases, with over one million new cases annually [2]. RA affects approximately 0.5–1% of adults [3]. RA is characterised by progressive inflammation, insidious pain, swelling, and early morning stiffness in one or a few joints, which progress to symmetrical polyarthritis. Joint function gradually deteriorates, progressing to irreversible joint damage and permanent loss of function. RA patients also experience extra-articular symptoms, such as fatigue, as well as several comorbidities, including cardiovascular disease (CVD), kidney disease, neurological manifestations, and pulmonary disease [4]. RA patients have increased mortality rates, primarily due to cardiovascular disease, respiratory diseases, and immune dysregulation. Unexpectedly, the burden of RA has increased significantly over the past 30 years and is predicted to continue rising. RA has been reported to have a 1.5–1.6-fold increase in standardised mortality ratios compared to the general population [3]. Infections and complications related to the cardiovascular and respiratory systems are assumed to be direct causes of death among RA patients [5]. Despite conflicting reports, the overall trend of increased mortality and morbidity remains dominant [3]. Therefore, progressive disability, an increased incidence, and mortality underscore the need to better understand RA risk factors and promoters, as well as develop effective treatments for the disease.

Several factors have been reported to induce RA, including genetic and environmental risk factors. Among these factors, family history, the *HLA-DRB1* gene, and several non-MHC genes are significant [1]. The susceptibility, persistence, and severity of RA depend on the complex interplay between the host and the environment; however, no clear cause has been identified [4,6]. For genetically predisposed individuals, environmental risk factors play a pivotal role in initiating and perpetuating RA [1]. Viral infections such as Epstein–Barr Virus (EBV) have been documented to play a role in the pathogenesis of RA [7]. The presence of human parvovirus B19 (B19V) has been associated with the progression and worsening of RA [8].

B19V, the smallest known DNA virus, is associated with various clinical diseases. With multiple receptors that bind tightly to human tissues, B19V can infect and cause multiorgan disease [9]. Transmission of B19V occurs through respiratory droplets, circulation, or transplacental transfer [10,11]. It is an undetected cause of various clinical manifestations, including hemolytic anemia, infectious erythema, arthropathy, non-specific polyarthralgia, and non-immune hydrops fetalis [9,12]. Rare manifestations, including neurological, hepatic, cardiac, and nephrological symptoms, have also been reported [12]. Fortunately, most B19V infections are asymptomatic or self-limiting [10]. Arthritis has been associated with viral infections such as B19V, HIV, alphaviruses, and hepatitis B and C viruses [13]. Among these, B19V is able to infect synovial tissue, activate innate immunity, and induce autoimmune responses through mechanisms such as molecular mimicry, immune complex deposition, and bystander activation [14]. Studies have documented B19V DNA persistence in the synovial membranes of RA patients. The virus targets immune cells in the synovium, causing infection and stimulating immune cells to secrete pro-inflammatory cytokines [8]. This suggests a potential role for B19V in RA pathogenesis [15]. RA patients who are serologically positive for anti-B19V-IgM typically experience an acute onset with prodromal symptoms and symmetrical polyarthritis of the small joints [16,17].

Although most cases of B19V infection are self-limiting, the virus has been reported as a factor that initiates and aggravates arthritis. However, there are limited reports on B19V infection and RA. The role of B19V in RA pathogenesis has not been fully investigated, nor has the B19V infection rate and immunological response in Vietnamese RA patients. This study investigated the prevalence of B19V DNA and anti-B19V antibodies and B19V genotypes in Vietnamese patients with RA and healthy individuals. It also examined the association between B19V infection and RA.

## 2. Materials and Methods

### 2.1. Patient Consent and Ethical Approval

All participants were informed of the study’s purpose, signed the consent form, and participated voluntarily. The study was approved by the Ethics Committee of Thai Nguyen Central General Hospital in Thai Nguyen, Vietnam (decision number 776/HĐĐĐ-BVTWTN, 29 October 2019). The study did not have any negative effects on the participants, including financial expenses, treatment plans, or health.

### 2.2. Data Collection and Study Design

From November 2019 to December 2021, the observational study recruited 115 hospitalised patients diagnosed with RA at the Department of Musculoskeletal System at Thai Nguyen National Hospital. Eligibility criteria for hospitalised RA patients (RA group) included a confirmed RA diagnosis according to the American College of Rheumatology (ACR) guidelines, being at least 18 years old, and providing informed consent. Eighty-six healthy controls (HC group) had no signs of RA or abnormal health. Specialists detected, diagnosed, and recorded the clinical and paraclinical data, as well as the confounding information such as chronic infections and medication use of the participants. There was no significant difference in gender or age between the RA and HC groups (*p* > 0.05).

### 2.3. RA Diagnosis and Clinical Evaluation Indices

Diagnosis of RA was according to the American College of Rheumatology (ACR-87) [18]. The clinical manifestation of patients was numbered by criterions including the visual analogue score (VAS) [19], the clinical disease activity index (CDAI) [20], the simplified disease activity index (SDAI) [21], the disease activity score (DAS) with 28-joint counts (DAS) [22,23], DAS using C-reactive protein (DAS 28-CRP) [24], DAS using ESR (DAS 28-ESR) [24], and the Steinbrocker classification of joint injury on X-Ray.

### 2.4. Detection of B19V DNA and DNA Sequence Analysis

The detection of B19V DNA in serum and synovial fluid by nested polymerase chain reaction (nested-PCR) using B19V-specific primers has been previously described [25]. Briefly, the primer pairs for the first PCR were as follows: sense (P5F) 5′-CAG TTT CGT GAA CTG TTA GT-3′ and antisense (P5R) 5′-ATT CCA CAA ATT GCT GAT ACA C-3′ (349 bp product). The primers used for the second PCR were sense (nP5F) 5′-CGT GAA CTG TTA GTT GGG GTT GA-3′ and antisense (nP5R) 50-AAT TGC TGA TAC ACA GCT TTA G-3′ (335 bp product). PCR conditions included an initial denaturation step of 94 °C for 5 min, followed by 35 cycles of denaturation at 94 °C for 30 s, annealing at 48 °C for 30 s, and extension at 72 °C for 45 s, followed by a final extension of 10 min at 72 °C [25]. Sample processing (DNA extraction, template preparation, spinning and aliquoting, and master-mix preparation) and PCR amplification were conducted in separate laboratory rooms using precautions to prevent assay contamination. The PCR products were analysed by electrophoresis on a 2% agarose gel and stained with ethidium bromide. All samples testing positive for B19 were confirmed with a quantification of real-time PCR (qPCR) using B19-specific primers as described previously [25,26]. Primer sequences used for qPCR were PVB1 (sense): 5′-GCT AAC TCT GTA ACT TGT AC-3′ and PVB2 (antisense): 5′-AAA TAT CTC CAT GGG GTTGAG-3′ (173 bp). B19 plasmid DNA of 3.5 × 10^2^ copies/mL of the WHO international standard for B19-DNA (NIBSC Code 99/800) was included as a positive control to exclude the possibility of cross-contamination. qPCR conditions included an initial denaturation step of 94 °C for 5 min, followed by 40 cycles of denaturation for 30 s at 94 °C, annealing for 30 s at 56 °C, and extension for 30 s at 72 °C, followed by a final extension of 10 min at 72 °C. Specificity of PCR products were also confirmed by automated DNA sequencing and B19V-DNA sequences were matched with the NCBI GenBank B19V-genome sequence (GenBank Accession No. AF162273).

### 2.5. Detect Anti-B19V Antibodies in Serum

Approximately 4 mL of peripheral whole blood was collected from participants in EDTA tubes. The tubes were then centrifuged at 5000 rpm for five minutes. The serum samples were stored at −80 °C until testing. Anti-B19V-IgG and -IgM were analysed using the Human Parvovirus B19 IgG ELISA Kit and the Human Parvovirus B19 IgM ELISA Kit (MYBioSource, Vancouver, BC, Canada). The Parvovirus B19V IgG/IgM ELISA Kit uses the solid-phase ELISA principle. The assays were performed strictly according to the manufacturer’s instructions. The sensitivity and specificity of the anti-B19V-IgG and anti-B19V-IgM were 100% and 84.4% and 100% and 95.5%, respectively.

### 2.6. B19V Genotype Analysis

The B19V genotypes were determined for B19V-DNA-positive samples. The B19V genome was analysed using direct sequencing with specific primers, nP5F and nP5R. The genetic sequence was constructed using Clustal W V. 2.1 and BLAST V. 2.17.0 (National Center for Biotechnology Information; http://www.ncbi.nlm.nih.gov/blast/). The sequence was analysed using BioEdit software V. 7.2 and presented in TreeView V2.0.8. Reference sequences were obtained from GenBank (https://www.ncbi.nlm.nih.gov/genbank/). These were genotype 1a (AB030694, DQ225150, M13178, DQ225148, DQ225149, DQ225151, AF162273, and AF113323), genotype 1b (DQ357065), and genotype 2 (AY044266, AY064475, AY064476, AY661664, and AY661663). Genotype 3 was represented by AX003421 and AY083234.

### 2.7. Statistical Analysis

Statistical descriptions and analyses were performed using Stata 18.0 (StataCorp, College Station, TX, USA). The distribution of all variables was determined via a skewness test. The difference between the two groups was analysed using an unpaired *t*-test or a Mann–Whitney test, depending on whether or not the variable followed a normal distribution. The association between two categorical variables was examined using a chi-square test or Fisher’s exact test. A binary logistic regression model adjusted for confounding factors (age and gender) was used to analyse the association between B19V DNA and B19V serology and clinical parameters of RA patients. Odds ratios (ORs) and 95% confidence intervals (CIs) were calculated. The difference was considered significant if *p* < 0.05.

## 3. Results

### 3.1. Clinical Manifestations of RA Patients

Table 1 describes the clinical characteristics of RA patients. All patients had joint pain ranging from mild to severe. Moderate pain was the most prevalent (53.91%), followed by severe pain (31.31%) and mild pain (14.78%). The prevalence of RA patients with and without knee effusion was 57.39% and 43.61%, respectively. Among all RA patients with morning stiffness, ≥1 h stiffness occurred 69.57% of the time, while <1 h stiffness occurred 30.43% of the time. Additionally, elevated CRP, RF, and anti-CCP levels were prevalent among RA patients, accounting for 90.43%, 85.22%, and 88.7% of cases, respectively. According to the Steinbrocker classification, stage 1 joint injury was the most prevalent (39.13%), followed by stage 2 (23.48%) and stage 3 (16.52%). The distribution of RA stages according to different international standards was also investigated. The results indicated that most RA patients had high DAS 28-CRP, DAS 28-ESR, CDAI, and SDAI scores. The majority of RA patients in this study had had the disease for less than five years (59.13%). The 5–10 years and >10 years groups accounted for 22.61% and 18.26%, respectively (Table 1).

### 3.2. The Prevalence of B19V Genome in RA Patients and Healthy Controls

**Figure 1:**
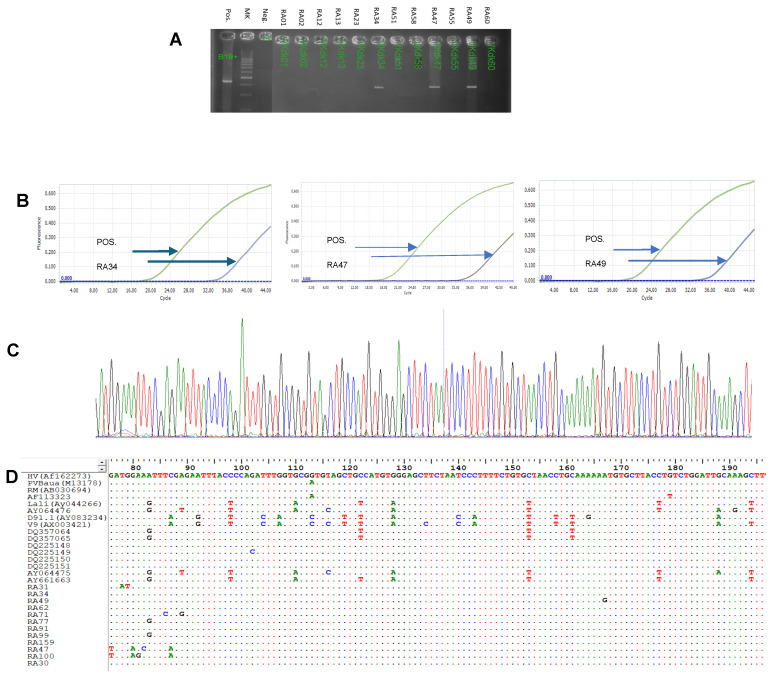
Detection of B19V genome in RA patients and healthy controls. (**A**) Electrophoresis images of positive and negative samples with B19V. (**B**) Real-time PCR image of positive samples with B19V. (**C**) Sanger sequencing image of positive samples. (**D**) Alignment of B19V sequences with reference sequences. Green line is positive control and the lines in other color are samples.

**Figure 2:**
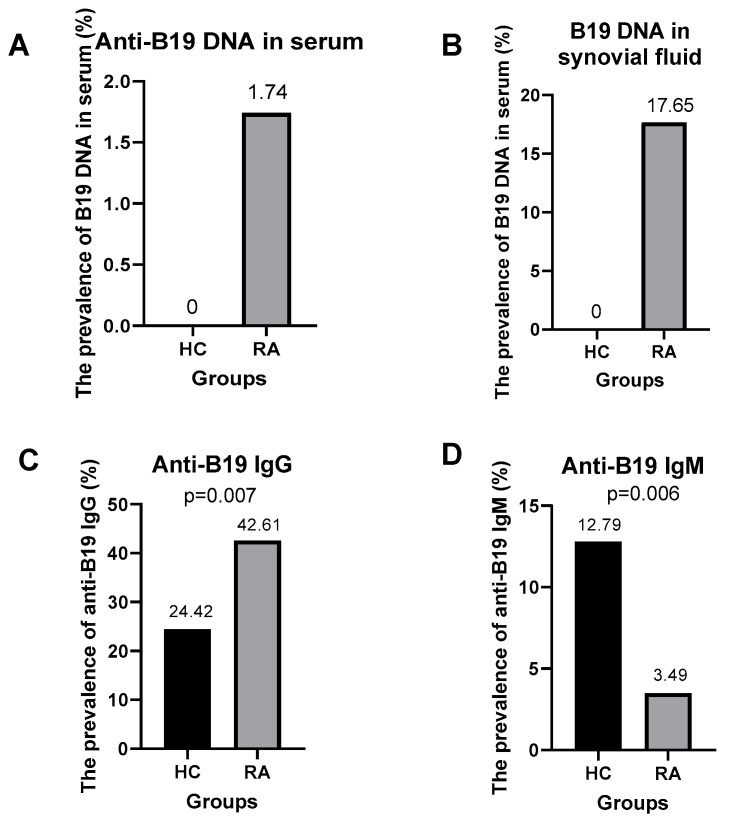
Prevalence of B19V infection in RA patients and healthy controls. (**A**) Percentage of B19V DNA positivity in serum. (**B**) Percentage of B19V DNA positivity in synovial fluid. (**C**) Percentage of anti-B19V IgG positivity. (**D**) Percentage of anti-B19V IgM positivity.

To examine the prevalence of B19V infection among RA patients and HCs, we tested for the presence of B19V DNA in serum and synovial fluid using nested-PCR to amplify the B19V NS1/VP1u region (Figure 1A) and quantitative PCR (qPCR) to amplify the B19V VP1/VP2 genes (Figure 1B). Positive B19V PCR amplicons were confirmed by direct DNA sequencing (Figure 1C). Analysis of the B19-NS1/VP1 amplicons revealed sequence variation differences among the B19V isolates to exclude cross-contamination of RA patient samples (Figure 1D). None of the HC group tested positive for B19V DNA (Figure 2A,B). Meanwhile, 2 out of 115 (1.74%) RA patients tested positive for B19V DNA in serum (Figure 2A). Interestingly, B19V DNA genomes were detected in 12/68 (17.65%) RA patients with knee effusion in synovial fluid (Figure 2B). A total of 14/115 (12.17%) RA patients had B19V DNA in either their serum or synovial fluid.

### 3.3. The Seropositive Rate of Anti-B19V IgM and IgG

We examined all serum samples from participants for anti-B19V IgG and anti-B19V IgM using immune assays. Anti-B19V IgG was detected in 21 out of 86 (24.42%) healthy subjects and 49 out of 115 (42.6%) RA patients. In contrast, anti-B19V IgM was detected in 3/115 (2.6%) RA patients and 11/86 (12.79%) healthy controls. Anti-B19V-IgG and/or anti-B19V-IgM were present in 51/115 (44.34%) RA patients and 22/86 (25.58%) HCs group (Table 2). The B19V infection status suggested chronic B19V infection predominance in RA patients (Figure 2C,D).

### 3.4. The Genetic Distribution of B19V Among Vietnamese RA Patients

**Figure 3:**
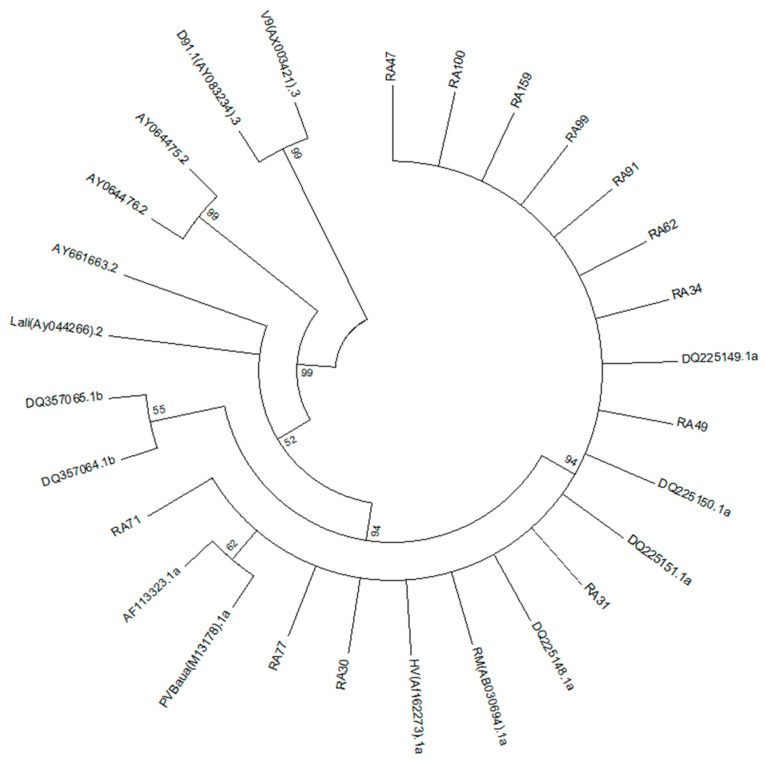
Phylogenetic analysis of 12 B19V isolates from synovial fluid of RA patients. The segments of B19V genome were amplified and sequenced by specific primers, nP5F and nP5R. The sequence was analysed using BioEdit software and constructed using Clustal W. Reference sequences for genotype 1a were AB030694, DQ225150, M13178, DQ225148, DQ225149, DQ225151, AF162273, and AF113323, for genotype 1b were DQ357065, for genotype 2 were AY044266, AY064475, AY064476, AY661664, and AY661663, and for genotype 3 were AX003421 and AY083234.

To determine the B19V genotype among Vietnamese RA patients, we sequenced the partial NS1/VP1 genes of B19V-positive samples. Phylogenetic analysis showed that all 14 B19V-positive samples (in both serum and synovial fluid) belonged to genotype 1 and subgenotype 1A. No B19V genotypes 2 or 3 were observed in RA patients (Figure 3).

### 3.5. B19V Infection Associated with RA Progression

To determine the frequency of B19V infection in RA progression, we analysed the association between B19V infection and RA patients and the HC group. The prevalence of B19 DNA was significantly higher in the serum and synovial fluid of RA patients than in the HC group (12.17% vs. 0%; Figure 2B). Interestingly, B19V genomes were frequently detected in synovial fluid (17.65%). Analysing the B19V serologic status, the prevalence of serum anti-B19-IgG was significantly higher in RA patients than in the HC group (42.60% vs. 24.42%, *p* < 0.01). However, the prevalence of anti-B19V-IgM was significantly higher in the HC group than in RA patients (12.79% vs. 2.60%, *p* < 0.01). In the univariate analysis, anti-B19V-IgG positivity was associated with an increased risk of RA (OR = 5.48; *p* = 0.011). Similarly, serum anti-B19V-IgM showed contrasting implications. The presence of anti-B19V-IgG and/or anti-B19V-IgM in serum was associated with an RA risk that was more than five times higher (adjusted OR = 5.15; *p* = 0.03) (Table 3).

## 4. Discussion

RA is a prevalent systemic autoimmune inflammatory disease that primarily affects the joints [1]. Progressive joint injury can lead to irreversible disability, posing a significant socioeconomic burden and reducing quality of life. In addition to complex inflammation and autoimmune responses, RA exhibits many extra-articular manifestations and comorbidities affecting the cardiovascular, respiratory, renal, gastrointestinal, ocular, cutaneous, and nervous systems [27,28,29]. RA has a female-dominated incidence, and gender is a well-known risk factor for the disease [3]. Women have a two- to threefold higher frequency of RA than men [14,30,31]. Consistent with this, our results showed a female dominance of 71.3%, compared to 28.7% male participants. Furthermore, our analysis of BMI revealed that 68.7% of RA patients had a BMI of 23 or less. There were no significant differences in BMI between the RA and HC groups, suggesting that being overweight or obese is not a major risk factor for RA in this cohort. However, obesity and being overweight have been shown to increase the risk of RA by 1.31 and 1.15 times, respectively [32]. Compared with normal-weight participants, overweight and obese RA patients had pooled relative risks (RRs) of 1.12 and 1.23, respectively [33]. Therefore, the role of BMI in RA among Vietnamese patients may differ from what has been observed globally.

Previous studies have shown that pathological RA is linked to several viral infections, including B19V [14,15]. B19V infection is widespread, occurring at varying rates depending on age. The highest rates are observed among school-aged children. A 16-year follow-up study in Serbia revealed an overall prevalence of 49.51% [34]. B19V seropositivity varies by age and gender. Significantly lower rates were observed in the paediatric cohort (9.12%) compared to the 40–59-year-old demographic (65.5%) [34]. In Brazil, the seroprevalence of anti-B19V IgG was highest in newborns (87%) and lowest in children aged four years and younger. It increased in the 31–40 age group [35]. A study of the B19V genome among German blood donors from 2015 to 2018 revealed a positivity rate of 0.013% [36]. Notably, a reemergence of B19V infection was detected in the Netherlands after the SARS-CoV-2 pandemic, with low detection rates in the general population and nearly absent cases in blood donors from 2009 to 2020 [37]. B19V infection has been reported in patients with juvenile chronic arthropathy, transfusion-dependent thalassemia, recurrent abortions, and leukaemia [9]. Previous studies have demonstrated significantly higher B19V infection rates in patients with hepatitis B virus (HBV) and *Plasmodium falciparum* malaria [38,39]. In this study, we compared the prevalence of B19V infection in healthy individuals to that in Vietnamese patients with RA. No B19V-DNA-positive cases were found in the serum of healthy controls, a significant decrease from the 4.7% (3/64) observed between 2000 and 2002 [39]. B19V DNA was detected in the serum of 2 out of 115 (1.74%) RA patients. Interestingly, B19V DNA was detected in the synovial fluid of 17.65% of the patients. In total, B19V DNA was detected in the synovial fluid or serum samples of 14 out of 115 RA patients, representing 12.17% of the studied group. This is the first report of B19V DNA positivity in the synovial fluid of RA patients in Vietnam, which suggests the presence of B19V infection in the joints. Our findings suggest a potential link between B19V and RA pathogenesis. Detecting B19V DNA in the synovial fluid of RA patients is significant because it indicates the virus’s presence in areas closely associated with the disease’s pathology. Synovial fluid is particularly important in RA because it lubricates the joints, and inflammation of the fluid contributes to the pain and stiffness characteristic of the disease. While a previous study found B19V DNA in a subset of RA patients, other studies have shown that B19V can be found in other types of arthritis, such as reactive arthritis and osteoarthritis, albeit less frequently [40]. This highlights the complexity of the relationship between B19V and different forms of arthritis. In this study, anti-B19V IgG seropositivity was found in 42.61% of RA patients, which is significantly higher than in the HC group (24.42%). Conversely, B19V IgM positivity was lower in RA patients (2.16%) than in HCs (12.49%). The high rate of B19V seropositivity in HCs suggests a risk of silent progression of B19V infection, as well as the potential for undetected cross-contamination. RA patients may have an impaired ability to eliminate acute B19V infection, leading to a significantly higher rate of anti-B19V IgG positivity compared to HCs. Immunosuppressive therapy for RA may also increase susceptibility to viral infections and impair viral clearance in RA patients [41]. In addition, we also found the absence of B19V DNA in healthy controls with detectable presence of IgG and IgM, indicating that the healthy individuals had cleared the virus and produced the antibodies for longer protection against B19V. These findings suggest that chronic B19V infection is prevalent among RA patients and that the presence of both anti-B19V IgG and B19V DNA is associated with RA in the Vietnamese population.

This study significantly contributes to our understanding of the role of B19V in the immunopathogenesis of RA in the Vietnamese population, addressing a gap in the global literature that has been scarcely covered. Through comprehensive logistic regression analyses, we revealed a complex, dualistic relationship between B19V serological and molecular markers (anti-B19V-IgG, anti-B19V-IgM, and B19V-DNA) and RA susceptibility. In the univariate analysis, prior exposure to anti-B19V-IgG positivity was associated with an increased RA risk (OR = 5.48; *p* = 0.011), suggesting a potential historical viral contribution to disease development. These findings underscore that neutralising antibodies may play a role in mitigating viral persistence and subsequent autoimmune activation. Active or recent B19V infection (as indicated by the presence of anti-B19V-IgM) may trigger or exacerbate autoimmune responses via mechanisms such as molecular mimicry, polyclonal B-cell activation, or chronic innate immune stimulation [41,42].

Understanding the genetic distribution is important for grasping the role of mutations due to cross-genotype infections. Three B19V genotypes are recognised globally, with genotype 1, particularly subgenotype 1A, being the most dominant worldwide [43]. Among Chinese patients with febrile rash illnesses, the prevalence of subgenotype 1A has been reported as 79.31% (23/29) [44]. In our previous analysis of HBV-infected individuals, we found a predominance of genotype 1 (96%) over genotype 2 (4%) [25]. In this study, we analysed the genetic distribution of a partial B19V genome in the Vietnamese RA population. Fourteen out of fourteen B19V-DNA samples (two serum and twelve synovial fluid samples) belonged to genotype 1 and subgenotype 1A. This indicates the dominance of the B19V genotype among Vietnamese RA patients. Despite the high rate of asymptomatic or self-limiting post-infection cases, B19V is known to cause arthritis onset and exacerbate arthritis symptoms [8,14,15]. B19V infection has also been associated with more severe clinical signs of malaria and hepatitis. Co-infection with B19V has been suggested as an aggravating factor in HBV-associated diseases and malaria [38,39]. While our analysis did not reveal a direct association between B19V infection and RA duration or clinical manifestations, the high prevalence of chronic B19V infection, especially in the synovial fluid of patients, underscores the importance of managing this infection.

Although this study revealed important findings, such as the association between B19V infection and RA progression and the distribution of B19V genotypes in Vietnamese RA patients, it has several limitations. First, the lack of control synovial fluid samples restricts our ability to assess the specificity of B19V detection in RA. Second, the small sample size and the single-centre design limit the generalisability and validation of the genotype distribution and overall findings. Third, as an observational study, it cannot establish a causal relationship between B19V infection and RA pathogenesis. Therefore, further studies, including longitudinal and functional studies, are needed to confirm these results and clarify the role of B19V in RA development. 

## 5. Conclusions

In conclusion, this study provides updated data on the high prevalence of B19V infection among Vietnamese RA patients. Our findings underscore the importance of considering B19V infection when managing RA, especially in patients presenting with unexplained joint effusion or elevated anti-B19V IgG seroprevalence. Clinicians should consider testing synovial fluid for B19V DNA, particularly in RA patients with persistent joint inflammation and effusion. Our results imply that viral factors, such as B19V, may contribute more significantly to joint damage and inflammation than previously recognised.

## Figures and Tables

**Table 1:** Clinical manifestations of RA patients.

Clinical Symptoms	Signs	N	%
Joint pain level	No pain	0	0
	Mild	17	14.78
	Medium	62	53.91
	Strong	36	31.31
Knee effusion	Positive	68	59.13
	Negative	47	40.87
Morning stiffness	≥1 h	80	69.57
	<1 h	35	30.43
CRP (mg/L)	Increased	104	90.43
RF (UI/L)	Increased	98	85.22
anti-CCP (UI/L)	Increased	102	88.70
Steinbrocker stages	No lesion	13	11.30
	Stage 1	45	39.13
	Stage 2	27	23.48
	Stage 3	19	16.52
	Stage 4	11	9.57
DAS 28_CRP	<2.6	1	0.87
	2.6–3.2	4	3.48
	3.2–5.1	55	47.83
	>5.1	55	47.83
DAS 28_ESR	<2.6	0	0
	2.6–3.2	1	0.87
	3.2–5.1	49	42.61
	>5.1	65	56.52
CDAI	<2.8	0	0
	2.8–10	7	6.1
	10–22	51	44.3
	>22	57	49.6
SDAI	<3.3	0	0
	3.3–11.0	1	0.87
	11.0–26	14	12.17
	>26	100	86.96
Disease duration (years)	<5	68	59.13
	5–10	26	22.61
	>10	21	18.26

CRP: C-reactive protein; RF: rheumatoid factor; Anti-CCP: anti-cyclic citrullinated peptide: DAS: Disease Activity Score; CDAI: Clinical Disease Activity Index; SDAI: Simplified Disease Activity Index.

**Table 2:** The serum anti-B19V status in RA patients and healthy controls.

Anti-B19V Status	HC (n = 86)	RA (n = 115)	Statistics
	n (%)	n (%)	
anti-IgG(+), −IgM(−)	21 (24.42)	49 (42.60)	OR = 2.31; 95%CI: 1.21–4.48; *p* < 0.01
anti-IgM(+),−IgG(−)	11 (12.79)	3 (2.60)	OR = 0.18; 95%CI: 0.03–0.78; *p* < 0.01
anti-IgG(+) and/or anti-IgM(+)	22 (25.58)	51 (44.34)	OR = 2.3; 95%CI: 1.19–4.49; *p* < 0.01

RA: rheumatoid arthritis; HC: healthy control; OR: odds ratio.

**Table 3:** Association of serum anti-B19V status with RA patients and healthy controls.

Anti-B19V Status	OR	95%CI	*p*
anti-IgG(+),−IgM(−)	5.48	1.48–20.29	0.011
anti-IgM(+), −IgG(−)	0.44	0.24–0.81	0.008
anti-IgM(+), and/or −IgG(+)	3.385	1.18–22.55	0.03 *

RA: rheumatoid arthritis; HC: healthy control; OR: odds ratio, * adjusted for age and gender.

## Data Availability

The datasets generated and/or analysed during the current study are not publicly available but are available from the corresponding author on reasonable request.
